# Human-Human Hand Interactions Aid Balance During Walking by Haptic Communication

**DOI:** 10.3389/frobt.2021.735575

**Published:** 2021-11-04

**Authors:** Mengnan Wu, Luke Drnach, Sistania M. Bong, Yun Seong Song, Lena H. Ting

**Affiliations:** ^1^ The Wallace H. Coulter Department of Biomedical Engineering, Emory University and Georgia Institute of Technology, Atlanta, GA, United States; ^2^ School of Electrical and Computer Engineering, Georgia Institute of Technology, Atlanta, GA, United States; ^3^ Mechanical and Aerospace Engineering, Missouri University of Science and Technology, Rolla, MO, United States; ^4^ Department of Rehabilitation Medicine, Division of Physical Therapy, Emory University School of Medicine, Atlanta, GA, United States

**Keywords:** haptic communication, walking balance, beam-walking, walking aid, balance assistance, human-human interaction, human-robot interaction, physical interaction

## Abstract

Principles from human-human physical interaction may be necessary to design more intuitive and seamless robotic devices to aid human movement. Previous studies have shown that light touch can aid balance and that haptic communication can improve performance of physical tasks, but the effects of touch between two humans on walking balance has not been previously characterized. This study examines physical interaction between two persons when one person aids another in performing a beam-walking task. 12 pairs of healthy young adults held a force sensor with one hand while one person walked on a narrow balance beam (2 cm wide x 3.7 m long) and the other person walked overground by their side. We compare balance performance during *partnered* vs. *solo* beam-walking to examine the effects of haptic interaction, and we compare hand interaction mechanics during partnered *beam-walking* vs. *overground walking* to examine how the interaction aided balance. While holding the hand of a partner, participants were able to walk further on the beam without falling, reduce lateral sway, and decrease angular momentum in the frontal plane. We measured small hand force magnitudes (mean of 2.2 N laterally and 3.4 N vertically) that created opposing torque components about the beam axis and calculated the interaction torque, the overlapping opposing torque that does not contribute to motion of the beam-walker’s body. We found higher interaction torque magnitudes during partnered beam-walking *vs*. partnered overground walking, and correlation between interaction torque magnitude and reductions in lateral sway. To gain insight into feasible controller designs to emulate human-human physical interactions for aiding walking balance, we modeled the relationship between each torque component and motion of the beam-walker’s body as a mass-spring-damper system. Our model results show opposite types of mechanical elements (active *vs*. passive) for the two torque components. Our results demonstrate that hand interactions aid balance during partnered beam-walking by creating opposing torques that primarily serve haptic communication, and our model of the torques suggest control parameters for implementing human-human balance aid in human-robot interactions.

## Introduction

Principles from human-human physical interaction may be necessary to design more intuitive and seamless robotic devices to aid human movement. Within a framework of interactive motor behaviors, haptic “collaboration” has been defined as when “both agents jointly try to develop a consensual solution to solve a problem” with symmetric behaviors of both agents seeking to reduce each other’s error and cost. Another class of joint behaviors is “cooperation,” which is an asymmetric relationship where “one agent focuses on itself and the other either obeys in the assistance or accepts to look for the other’s task in the education” (Jarrassé et al., 2012). A robotic device that aids a person’s walking falls under the class of “cooperation,” where the robot seeks either to assist or educate the human. Hand-operated robotic devices have recently been developed to aid walking similar to a powered cane (Suzuki et al., 2009; Di et al., 2016; Nakagawa et al., 2016; Lam and Fujimoto, 2019; Trujillo-León et al., 2020) or walker/rollator (see reviews in (Martins et al., 2012, 2015; Werner et al., 2016)). Robotic walking aids have been designed to aid balance through a variety of methods such as providing mechanical support during falls (Hirata et al., 2006; Suzuki et al., 2009; Mou et al., 2012; Geravand et al., 2015; Di et al., 2016; Lam and Fujimoto, 2019), preventing risky postures (Nakagawa et al., 2016), or providing a proprioceptive cue (Stramel et al., 2019), but it is unclear which strategies are most intuitive and beneficial to the user. Meanwhile, a person can quickly and intuitively aid another person’s balance during walking by holding their hand. Greater understanding of the mechanics of human-human balance aid may establish principles to guide improved control laws for robotic devices.

Previous studies on light touch measured forces at the hands to examine the how physical interactions aid balance. Early work on light touch, originally defined as forces <1 N, showed that postural sway during standing balance was reduced when a person used a fingertip to touch a stationary object; this reduction in postural sway was interpreted as improved balance control due to more sensitive sensory feedback for maintaining upright posture through light touch, and not due to mechanical support (Holden et al., 1994; Jeka and Lackner, 1994). Later studies showed that light touch between two persons can also decrease postural sway (Johannsen et al., 2009, 2012, 2017, 2018; Reynolds and Osler, 2014; Steinl et al., 2018). Few studies have examined light touch during walking, and then only between a human and an object or device. Some results show that light touch with an object/device can improve walking balance either on a treadmill or overground, as measured by reduced body sway (Dickstein and Laufer, 2004; Forero and Misiaszek, 2013), pelvic acceleration (Boonsinsukh et al., 2009), decreased step width (IJmker et al., 2015; Stramel et al., 2019), and lower margin of stability variance (Oates et al., 2017). However, we are not aware of any previous work examining whether light touch between two persons can also improve walking balance.

Perhaps engaging similar sensorimotor mechanisms as light touch, haptic communication has been used to explain the benefits of human-human hand interactions for performing constrained physical tasks, but mostly during upper-limb manipulation. Human dyads have been shown to perform better than the worse partner in reaching (Reed et al., 2006; Reed and Peshkin, 2008), object manipulation (van der Wel et al., 2011; Mojtahedi et al., 2017; Jensen et al., 2021), target tracking (Wegner and Zeaman, 1956; Glynn and Henning, 2000; Feth et al., 2009; Ganesh et al., 2014; Melendez-Calderon et al., 2015), and force tracking (Masumoto and Inui, 2013). In studies that measured each person’s force or torque contribution independently through coupled robotic devices, human partners have been shown to exert opposing forces or torques that cancel each other (Reed et al., 2006; van der Wel et al., 2011; Madan et al., 2015; Melendez-Calderon et al., 2015; Mojtahedi et al., 2017). These opposing forces/torques do not contribute to motion of the object being manipulated, and have been proposed to create a haptic communication channel that facilitates interpersonal collaboration (van der Wel et al., 2011; Madan et al., 2015; Jensen et al., 2021). As it consists primarily of information transfer (van der Wel et al., 2011), haptic communication may describe the same phenomenon as light touch.

Previous work measuring hand forces between two persons during walking provide an estimate of the range of forces involved in passive dynamics and haptic communication during walking. Small ranges of hand contact forces (2–4 N) have been measured between two persons walking side-by-side on separate treadmills (Sylos-Labini et al., 2018), a task that does not involve explicit movement goals, and thus the force magnitudes likely characterize dynamics of arm motion and the hand connection. Larger, but still small peak forces (10–30 N) were sufficient to convey movement goals and timing between partners with assigned roles of leader and follower in the absence of visual or audio feedback (Sawers et al., 2017). Similar peak force levels (up to 8.4 N) were measured for two persons walking and carrying a large object together (Jensen et al., 2021), another physical interaction task with a defined goal. However, no previous work has measured hand forces used to aid balance during walking.

A suitable paradigm for examining physical interactions that aid balance must appropriately challenge balance in order to elicit aid. As individuals without balance impairments are unlikely to experience significant balance challenge during overground walking, beam-walking has been used as an experimental task to challenge balance in unimpaired adults (Speers et al., 1998; Domingo and Ferris, 2009, 2010; Sipp et al., 2013; Sawers and Ting, 2015; Chiovetto et al., 2018). Furthermore, Sawers and Ting 2015 showed that the distance walked on medium and narrow-width beams can distinguish between expert, novice, and balance-impaired populations, leading to the use of this task as a clinical or laboratory tool for measuring dynamic balance. Here we take advantage of a challenging beam-walking task to evaluate whether and how hand forces between two persons aid balance.

The current study examines the effects of human-human physical interaction to aid balance in a beam-walking task. Pairs of unimpaired young adults held the ends of a custom force handle device with one hand while one person walked on a narrow balance beam and the other person walked overground by their side. We compared balance performance during partnered *vs.* solo beam-walking to examine the effects of haptic interaction, and we compared hand interaction mechanics during partnered beam-walking *vs.* overground walking to examine how the interaction aided balance. We calculated the torque components on the beam-walker’s body generated by hand forces and the interaction torque, overlapping and opposing torque that does not contribute to motion of the beam-walker’s body. Defining haptic communication as exchange of information through sensory feedback elicited from physical contact (i.e., touch), we hypothesize that balance is aided through haptic communication and predict that interaction torque is greater for partnered beam-walking than partnered overground walking. In order to gain insight into feasible controller designs to emulate human-human physical interactions for aiding walking balance, we further model the relationship between torques and the beam-walker’s angular sway as a second order system. Our approach examines physical partner interactions in a paradigm relevant to assistance and rehabilitation (i.e., the beam-walking task challenges balance in unimpaired young adults while overground walking challenges balance in impaired populations), and our results suggest haptic interaction parameters that can be implemented in robotic devices to aid balance.

## Materials and Methods

### Participants

We measured kinematics and hand forces in 12 pairs of healthy young adults. (4 male/20 female, 25.1 ± 3.2 years old, 167.2 ± 7.3 m height, 66.8 ± 10.8 kg weight). For all participants, exclusion criteria were medical conditions, assessed by self-report, that could result in impaired balance or sensory loss, including significant musculoskeletal, neurologic, or cardiopulmonary conditions. Participants were assigned to pairs and assigned roles of “beam-walker” or “partner” within each pair. The Institutional Review Board of Emory University approved all protocols. Data was collected during a single session.

### Setup and Protocol

**FIGURE 1:**
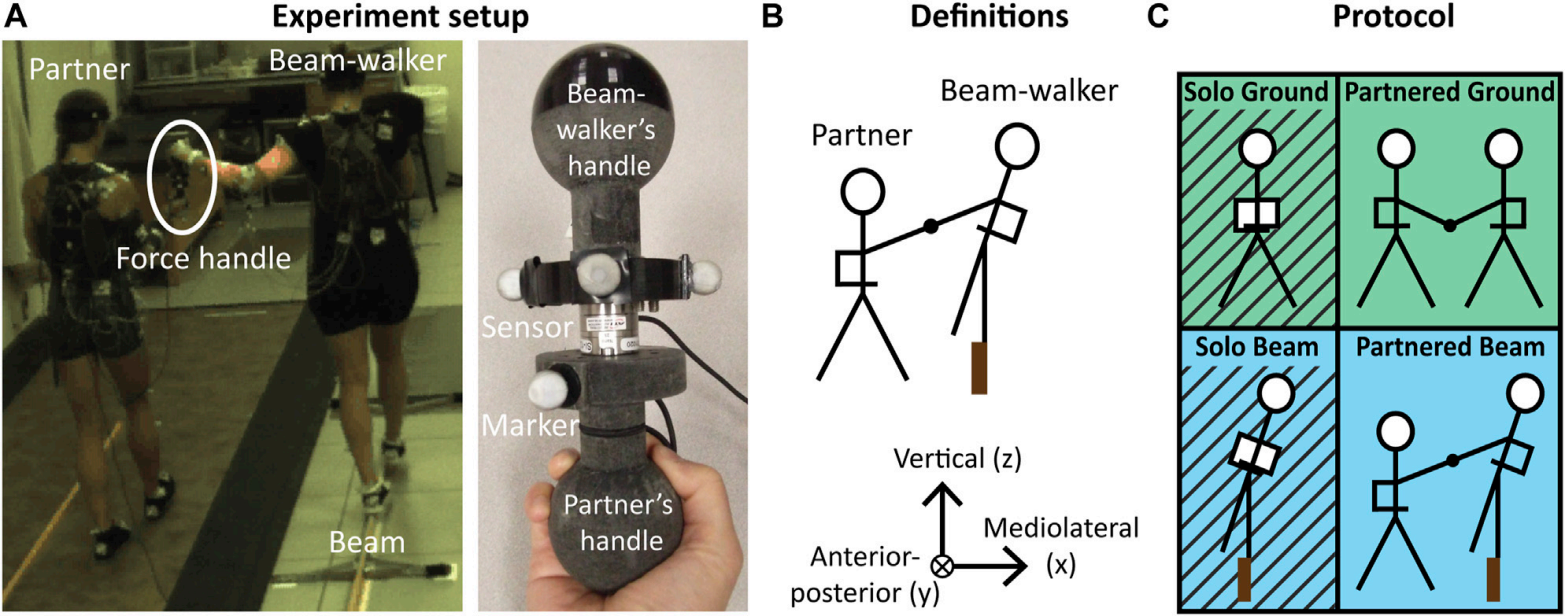
Experiment setup and protocol. **(A)** partner and beam-walker during partnered beam-walking condition (left) and close-up of custom force handle device with sensor and motion capture markers (right). **(B)** Coordinate frame definition. **(C)** Experiment protocol compares Solo vs. Partnered Beam-walking to examine effects of assistance on balance performance and Partnered Overground vs. Beam walking to examine the mechanics of assistance.

During the partnered beam-walking condition, the beam-walker walked on a narrow balance beam (2 cm wide x 3.7 m long, the same narrow beam as in Sawers and Ting 2015) while the partner walked overground on the beam-walker’s left side (Figures 1A,B). Each person held one end of a custom handle device with a force-torque sensor (ATI Nano25) in the center that measured hand interaction forces (Figure 1A). The beam-walker began the trial with their right foot on the beam and their left foot on the ground. After the “go” signal, the beam-walker lifted their left foot from the ground and began walking. The beam-walker was required to maintain their left arm flexed at the elbow but not touching the body, hold the top side of the force handle “like a computer mouse,” and keep their right arm across their stomach in order to maintain a consistent effect of arm posture and remove effects of arm motion on balance. The partner was instructed to maintain the mirrored arm posture as the beam-walker, and to hold the bottom side of the force handle “like an Olympic torch”. The beam-walker was instructed to stop if they uncrossed their right arm or stepped off the beam. No explicit instructions were given regarding stepping pattern or walking speed. The partner was instructed to follow the beam-walker’s speed while providing their right hand as assistance.

The control conditions consisted of solo overground walking, solo beam-walking, and partnered overground walking (Figure 1C). For the solo walking conditions, the beam-walker held the force handle and maintained the same arm posture as in partnered beam-walking. During partnered overground walking, the beam-walker and the partner both held the force handle and maintained arm postures similar to partnered beam-walking. For all overground walking conditions, participants walked forward at a preferred speed without further instructions. 10 trials were completed for each condition.

Both the beam-walker and partner were instrumented with a full-body Plug-in Gait marker set. Kinematic data was recorded at 120 Hz by a ten-camera motion capture system (Vicon Nexus) and force data was recorded at 1,200 Hz.

### Data Analysis

Motion capture marker data was median-filtered and low-pass filtered (bidirectional 3rd order Butterworth, cutoff frequency of 10 Hz) in Matlab (Mathworks, Natick, MA,United States). Force data was downsampled to match marker data sampling frequency and then low-pass filtered (bidirectional 3rd order Butterworth, cutoff frequency of 10 Hz) so as not to introduce any lag. Forces were aligned to the marker data coordinate frame system using the positions of markers placed on the custom handle. We averaged metrics across all 10 trials of a given condition for each partnership.

#### Balance Performance

We quantified improvement in balance performance for solo *vs.* partnered beam-walking using kinematic marker data. Previous studies have shown the validity of distance completed on the beam (Sawers and Ting, 2015), standard deviation of lateral body sway (Domingo and Ferris, 2009, 2010), and angular momentum about the beam (Chiovetto et al., 2018) to characterize balance performance during human beam-walking. The time window of analysis was determined by the period starting when the beam-walker’s left heel’s vertical displacement reached its first peak and ending when the beam-walker reached the end of the beam or fell off the beam. As a metric of overall performance in a trial, the distance completed along the beam by the beam-walker was calculated from the anterior-posterior displacement of the torso marker (clavicle or C7 marker for cases where the clavicle was obstructed). As a metric of instantaneous balance, sway variability was calculated as the standard deviation of the mediolateral position of the beam-walker’s torso marker. As another metric of instantaneous balance, angular momentum of the beam-walker about the reference point was calculated as Ly = I*ω, where I = inertia of the beam-walker’s body approximated by a thin rod with mass = body weight and length = height, and ω = angular velocity of the torso marker about the reference point. The reference point was located at either the intersection of the beam axis with the frontal plane for beam-walking trials or the mean mediolateral position of the beam-walker’s torso marker at ground height for overground walking trials. The RMS mean of angular momentum was calculated per trial.

To characterize the effect of haptic interaction on balance, we compared balance performance metrics between solo and partnered beam-walking. Since all beam-walkers completed the full beam length in all partnered beam-walking trials, the distance completed during the solo beam-walking condition was compared against the full beam length. A Student’s t-test was used as the distance completed data were normally distributed. Non-parametric Wilcoxon sign-rank tests were used for sway variability and angular momentum metrics as data were not normally distributed.

We performed additional pairwise comparisons on sway variability and angular momentum metrics to confirm that a partner did not affect balance for overground walking and that the beam-walking task challenged balance. First we tested if the metrics were significantly different during *solo* overground vs. *partnered* overground walking. Then we tested if the metrics were significantly different during solo *overground walking* vs. solo *beam-walking*. Student’s t-tests were used as the data compared were all normally distributed.

We also performed correlation analysis on balance performance improvement and solo beam-walking ability to test whether lower-skilled participants benefitted more from haptic interaction than higher-skilled participants. We used the distance completed on the beam during the solo beam-walking trials as a metric of solo balance ability and difference (between solo and partnered beam-walking) in sway variability as a metric of balance performance improvement. Pearson’s correlation was used as data were normally distributed.

#### Interaction Mechanics

**FIGURE 2:**
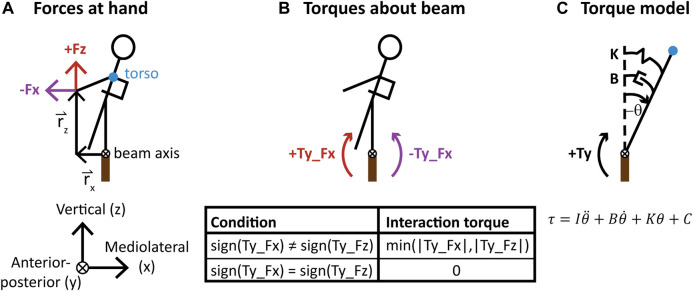
Torque on the beam-walker’s body about the beam axis (the midline of the beam points into the page) due to hand interaction forces. **(A)** Torque components were calculated by multiplying each force component by its moment arm (r), defined as the distance from the beam axis to the beam-walker’s left finger marker in the frontal plane. Blue dot indicates torso marker. **(B)** The lateral (x) and vertical (z) hand force components create opposing torques in the frontal planes, and the interaction torque is defined in the table. **(C)** A mass-spring-damper model is fit between the beam-walker’s angular torso state and each torque component (Ty_Fx and Ty_Fz) separately. Blue dot indicates torso marker.

To examine the mechanics of haptic interaction to aid balance, we measured lateral and vertical hand forces between the partners and calculated the torques created by these forces on the beam-walker’s body about the beam axis (Figures 2A,B). We focused on torques about the beam axis, located at the midline of the beam, based on previous work that showed beam-walking can be more simply analyzed by angular momentum in the frontal plane compared to segmental kinematics, and that the most salient reference frame for rotational dynamics is the beam axis (Chiovetto et al., 2018). To preprocess force data, we first subtracted off a zero-load voltage bias value per experiment session to account for drift of the sensor across sessions and then used the position data of motion capture markers placed on the sensor to obtain forces in the same coordinate frame as kinematic data. Torque components were calculated by multiplying each force component by its orthogonal moment arm, defined as the distance from the beam axis to the beam-walker’s left finger marker in the frontal plane (Figure 2A). Given that forces and torques oscillated over the course of a trial, we calculated standard deviation to characterize the magnitude of haptic interaction (Figures 4A, 5A). For torques, we also calculated the mean over a trial to characterize the direction (clockwise *vs*. anticlockwise in the frontal plane) of haptic interaction. Since torques due to lateral and vertical hand forces were often in opposing directions, we calculated the interaction torque as the amount of overlap in opposing torques at a given time (Figure 2B). This calculation of interaction torque is based on the definition of interaction forces in linear human-human interaction tasks (Groten et al., 2009; Madan et al., 2015).

To characterize the dynamics of physical interaction, we performed statistical comparisons between partnered overground walking and partnered beam-walking. The metrics analyzed were standard deviation of each component of force and torque, mean of each component of torque, and standard deviation of interaction torque. We used paired t-tests or sign-rank tests when data were non-normal. We also performed correlation analysis between balance performance improvement (difference between solo and partnered beam-walking sway variability) and torque metrics (standard deviation of net torque, Ty_Fx, Ty_Fz, and interaction torque) to further test how interaction dynamics relate to balance. We used Pearson’s correlations as all data were normally distributed. Due to data collection errors, only data from nine pairs of participants were usable for dynamics analysis.

#### Model of Relationship Between Torque and Body Motion

To better understand the mechanics of haptic interaction to aid balance and extract parameters that can potentially be used in design of human-robot interaction, we modeled the relationship between torque on the beam-walker’s body about the beam axis and the angular state of the beam-walker’s torso as a mass-spring-damper system (Figure 2C). We performed linear regression on each component of torque separately as the forces created opposing torques. Angular displacement (θ) of the torso was calculated as the deviation from vertical of a vector from the beam axis to the beam-walker’s torso marker (Figure 2C). The derivative of the filtered (bidirectional 3rd order Butterworth, cutoff frequency of 12 Hz) angular displacement was used to obtain angular velocity ( $\dot{\theta }$ ), and this signal was filtered again (same filter parameters as for displacement data) before differentiating to obtain angular acceleration ( $\ddot{\theta }$ ). The regression algorithm for each trial consisted of 1) regressing to all three torso state terms (displacement, velocity, and acceleration), 2) discarding any non-significant terms, as defined by regression coefficients with confidence intervals that included zero, and repeating steps 1) and 2) until only significant terms or no terms remained in the final regression model. If the final regression model reached statistical significance (*p* < 0.05), the coefficients from the trial were included in calculations of the average coefficient values for the partnership. The R^2^ value of the final model was calculated to measure the quality of fit for each trial.

We performed *t*-tests to compare if values of each coefficient from the mass-spring-damper model were significantly different from zero. We chose sign conventions for the model such that coefficients with positive values correspond to passive mechanical elements that resist motion of the beam-walker’s body while negative values correspond to active elements that amplify motion. (Figure 2C equation).

## Results

### Balance Performance

**FIGURE 3:**
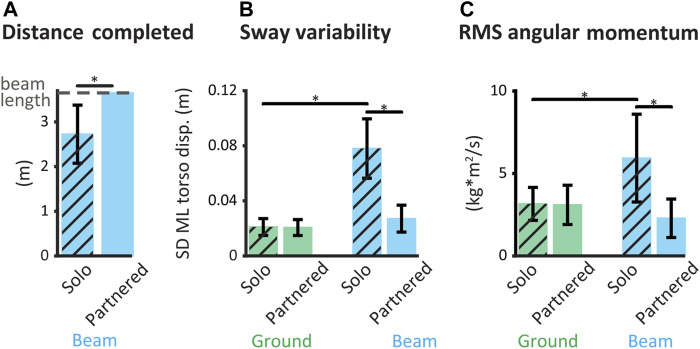
Balance performance metrics in solo and partnered beam-walking. *t-test *p* < 0.05. Error bars represent ± one standard deviation. **(A)** Mean beam distance completed per trial. All partnerships completed the entire beam length (dashed line) on every partnered beam-walking trial. **(B)** Mean sway variability of beam-walker’s torso. **(C)** Mean RMS angular momentum of beam-walker’s body about the beam axis.

In all beam-walkers, balance performance during beam-walking improved with a partner (Figure 3). The mean distance completed during solo beam-walking was 2.7 ± 0.65 m whereas every beam-walker completed the entire beam length (3.7 m) during every partnered beam-walking trial (*p* = 0.001) (Figure 3A). Across participants, median sway variability decreased 67% from solo (0.077 ± 0.022 m) to partnered (0.025 ± 0.025 m) beam-walking (*p* = 0.002) (Figure 3B). Median angular momentum of the beam-walker’s body about the beam axis decreased by 64% from solo (6.4 ± 2.7 kg* m^2^/ s) to partnered (2.3 ± 1.2 kg* m^2^/ s) beam-walking (*p* = 0.002) (Figure 3C).

Hand interactions with a partner did not affect balance for overground walking, and the beam-walking task challenged balance in the solo participant (Figure 3). Both sway variability and angular momentum means showed no significant difference between *solo* overground walking *vs.* *partnered* overground walking (*p* = 0.74 and 0.55, respectively). Both sway variability and angular momentum means were smaller during solo *overground* walking *vs.* solo *beam-walking* (*p* < 0.001 and *p* = 0.001, respectively).

Improvement in balance performance with a partner was not correlated with solo balance ability. There was no correlation between reduction in sway variability and solo beam distance completed (*p* = 0.31). There was also no correlation between reduction in angular momentum and solo beam distance completed (*p* = 0.83).

### Interaction Mechanics

**FIGURE 4:**
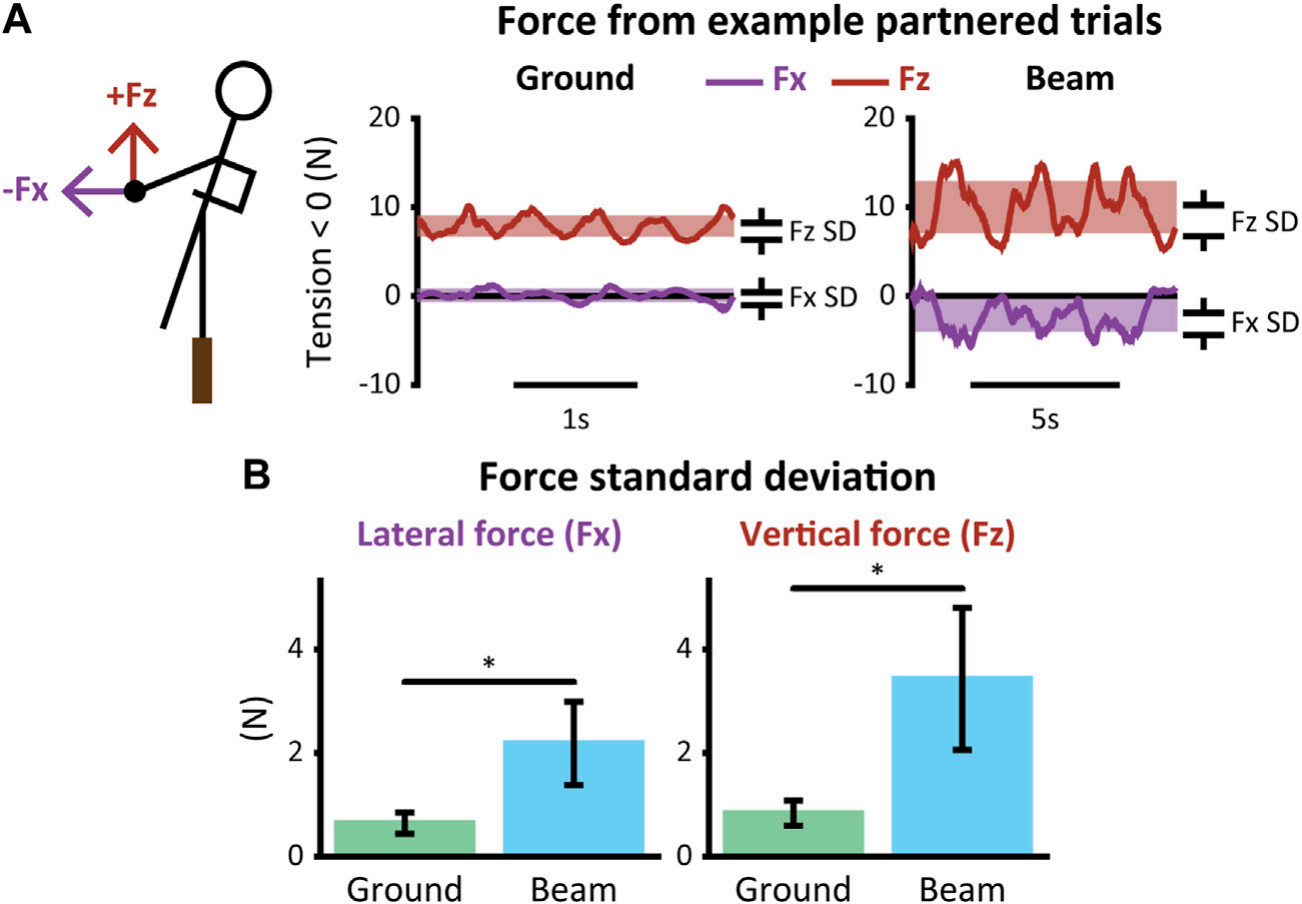
Hand forces during partnered walking. *t-test *p* < 0.05. Error bars represent ± one standard deviation. **(A)** Lateral (Fx) and vertical (Fz) hand forces during example trials of overground (left) and beam (right) walking. Standard deviation was calculated per trial to quantify magnitude of balance assistance. **(B)** Standard deviation of force in each direction during overground and beam walking for all participants.

Hand forces were small overall but higher during partnered beam-walking than partnered overground walking (Figure 4). In general, lateral and vertical hand forces oscillated about a mean value over the course of each trial (Figure 4A). Hand force standard deviation in both the lateral (Fx) and vertical (Fz) directions were higher during partnered beam-walking (Fx = 2.2 ± 0.80 N, Fz = 3.4 ± 1.4 N) than partnered overground walking (Fx = 0.64 ± 0.20 N, Fz = 0.84 ± 0.24 N; *p* < 0.001) (Figure 4B).

**FIGURE 5:**
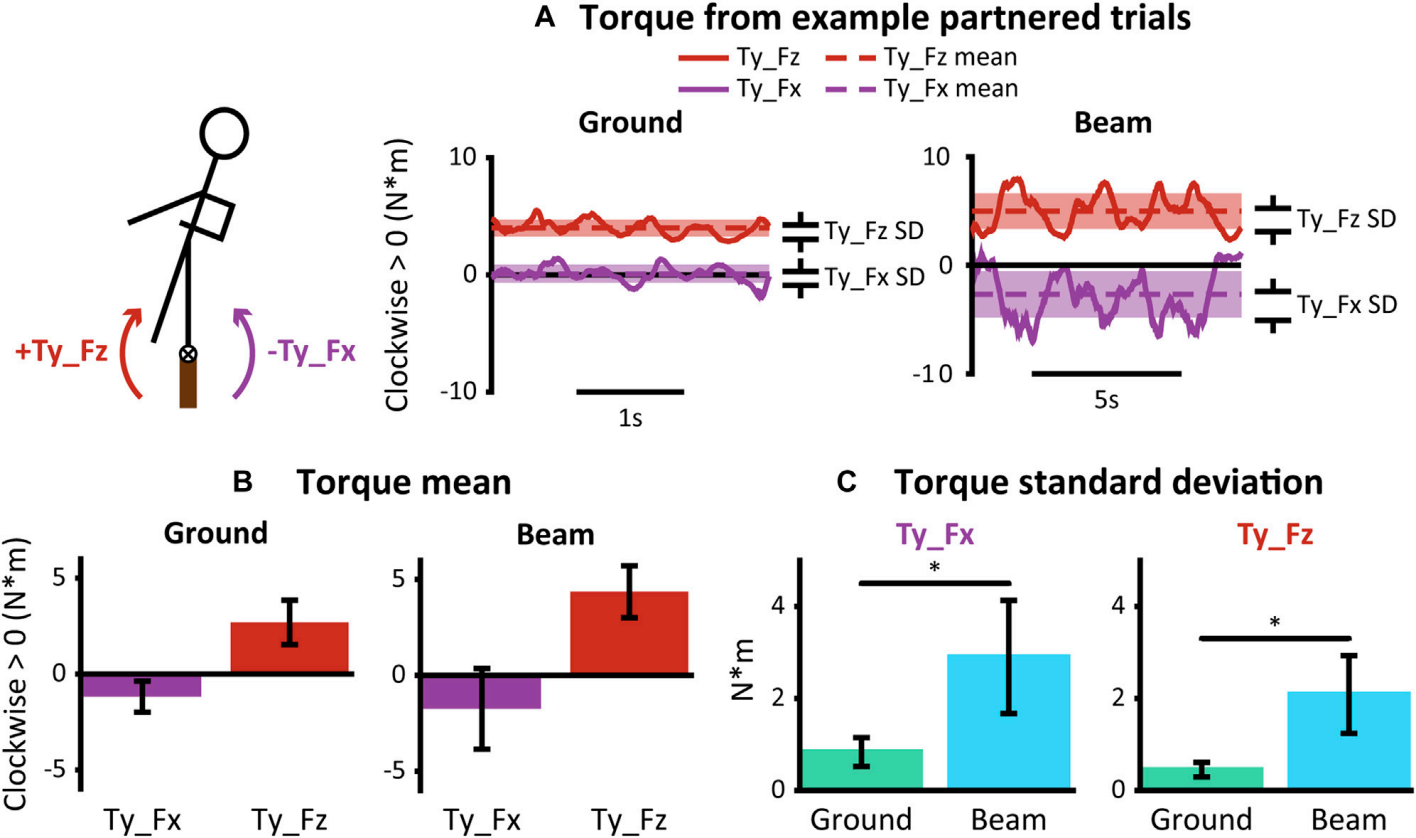
Torques on beam-walker’s body due to hand forces during partnered walking *t-test *p* < 0.05. Error bars represent ± one standard deviation. **(A)** Torques due to lateral (Ty_Fx) and vertical (Ty_Fz) hand forces during example trials of overground (left) and beam (right) walking. Standard deviations were calculated per trial to quantify magnitude of balance assistance. Means were calculated per trial to distinguish directionality. **(B)** Torque means for all participants. Means for Ty_Fx and Ty_Fz were different from zero and opposite in sign for both overground (left) and beam (right) walking. **(C)** Torque standard deviations for all participants.

The mechanical effect of lateral and vertical hand forces was to create torques about the beam with opposing mean directions and oscillations that were greater during partnered beam-walking *vs*. partnered overground walking (Figure 5). In example partnered walking trials, torques oscillated in an opposing manner (Figure 5A). Torques from Fx had negative means that were different from zero (Ground: −1.2 ± 0.81 N*m, *p* = 0.002; Beam: −1.7 ± 2.1 N*m, *p* = 0.04) while torques from Fz had positive means that were different from zero (Ground: 2.7 ± 1.2 N*m, *p* < 0.001; Beam: 4.4 ± 1.4 N*m, *p* < 0.001) (Figure 5B). Standard deviation of torques were higher during partnered beam-walking (Ty_Fx = 2.8 ± 1.2 N*m, Ty_Fz = 2.1 ± 0.85 N*m) than partnered overground walking (Ty_Fx = 0.84 ± 0.31 N*m, Ty_Fz = 0.45 ± 0.16 N*m) (*p* = 0.001 for Ty_Fx and *p* < 0.001 for Ty_Fz) (Figure 5C).

**FIGURE 6:**
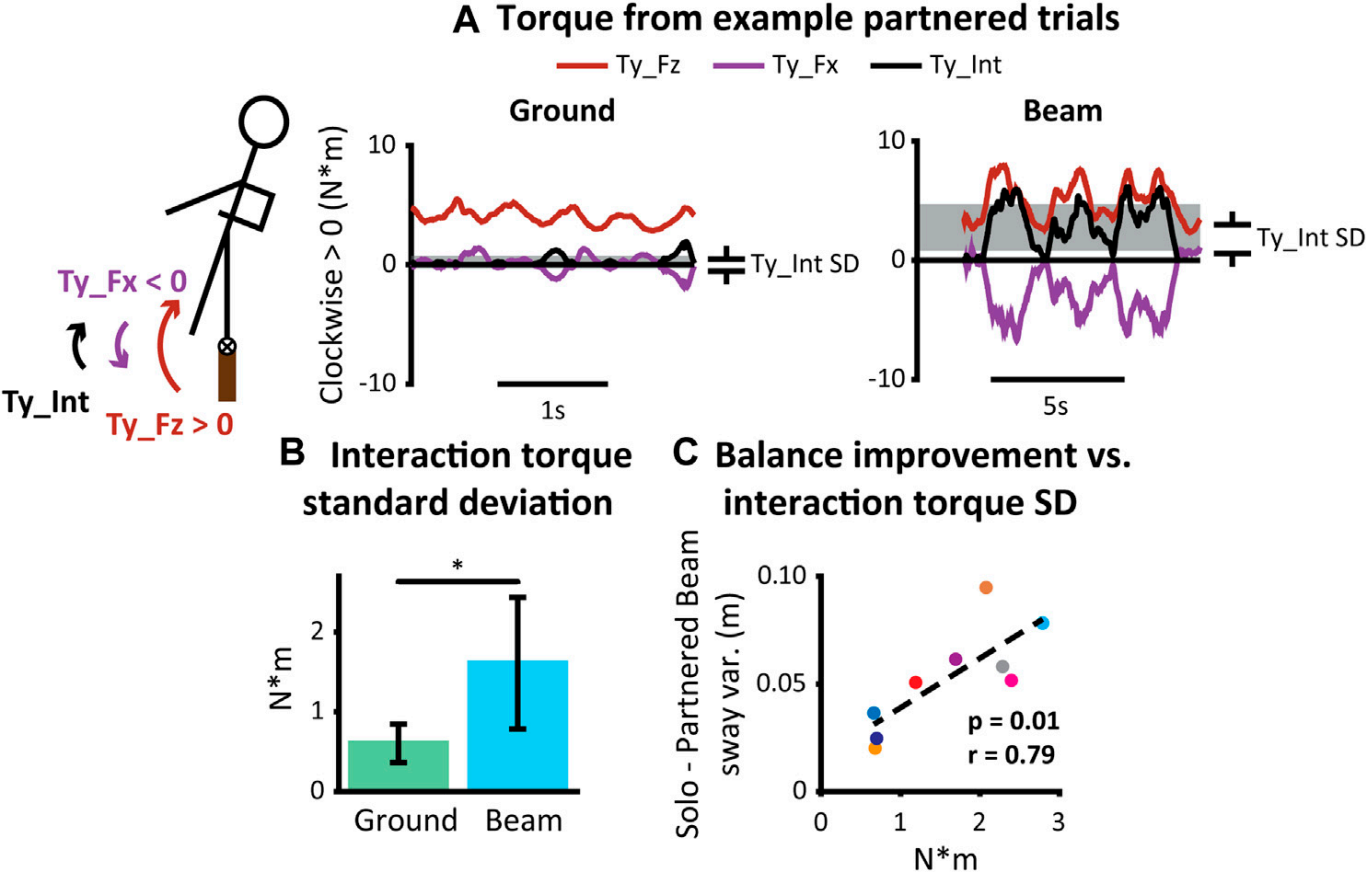
Interaction torques during partnered walking. *t-test *p* < 0.05. Error bars represent ± one standard deviation. **(A)** Interaction torque, the overlapping and opposing torque due to lateral (Ty_Fx) and vertical (Ty_Fz) hand forces, during partnered beam-walking were oscillatory over the course of a trial. Standard deviation was calculated to quantify magnitude of interaction torque. **(B)** Interaction torque was higher during partnered beam-walking than partnered overground walking. **(C)** Greater interaction torque standard deviation correlated with greater improvement in balance performance. A different color dot denotes each partnership.

Balance improvements during partnered beam walking were associated with the standard deviation of interaction torque (Figure 6). Torques due to lateral and vertical hand forces generally oscillated in a manner that oppose each other (Figure 6A). The amount of interaction torque was higher during partnered beam-walking than partnered overground walking (1.6 ± 0.83 N*m *vs*. 0.60 ± 0.24 N*m, *p* = 0.003) (Figure 6B). Greater standard deviation of interaction torque was also correlated with greater improvement in balance performance (*p* = 0.01, Pearson’s *⍴* = 0.79) (Figure 6C). There were no correlations between any of the other torque metrics (standard deviation of net torque, Ty_Fx, and Ty_Fz) and balance improvement (*p* > 0.05).

### Model of Relationship Between Torque and Body Motion

**FIGURE 7:**
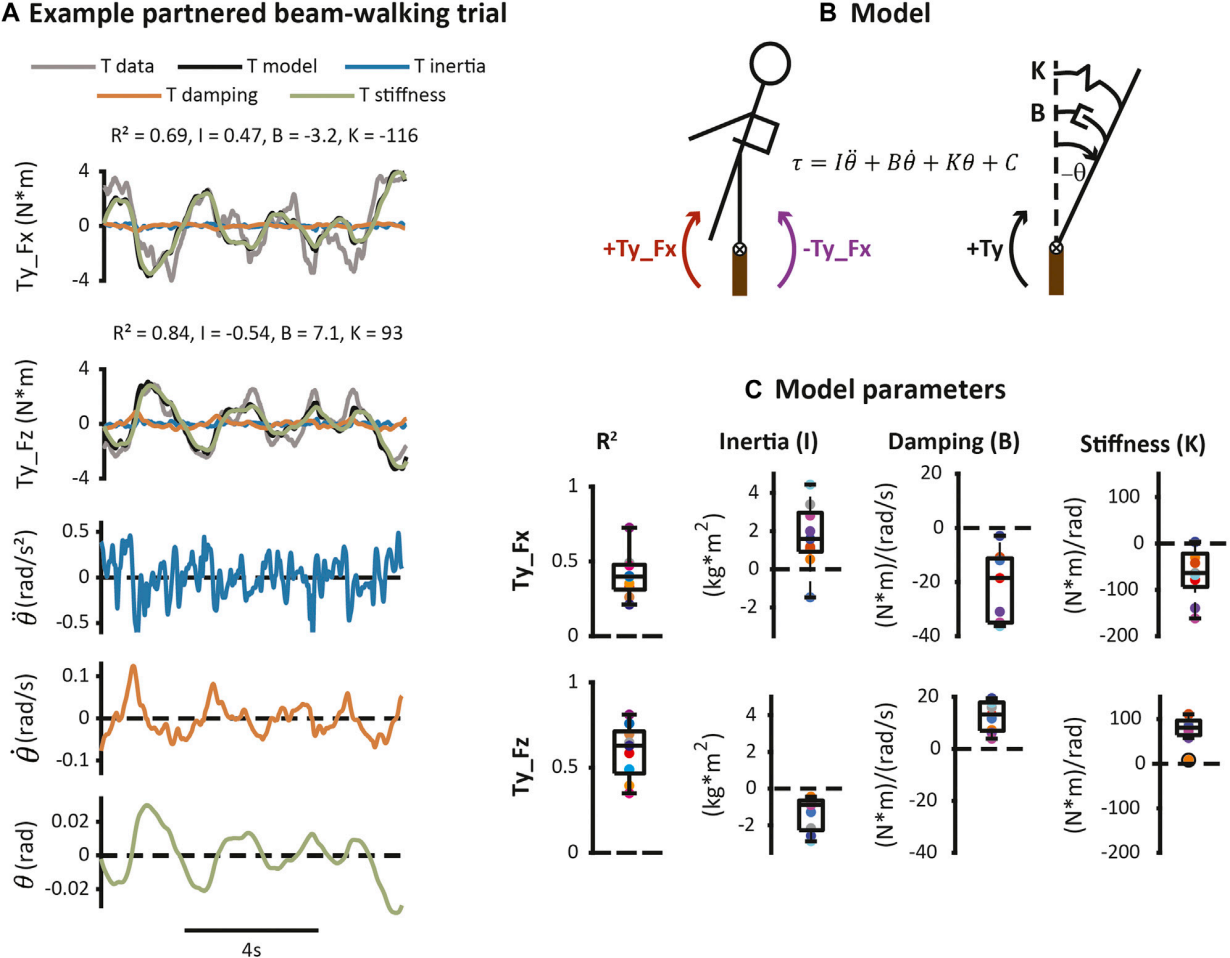
Model of relationship between torque and beam-walker’s body motion. Error bars represent ± one standard deviation. **(A)** Example data from partnered beam-walking trial. **(B)** A mass-spring-damper model was fit between the beam-walker’s angular torso state and each torque component (Ty_Fx and Ty_Fz) separately. **(C)** Coefficients of mass-spring-damper model. Top row corresponds to torque due to lateral hand forces (Ty_Fx), and bottom row corresponds to torque due to vertical hand forces (Ty_Fz). Columns show values of quality of fit, inertia, damping, and stiffness. Boxplots show group data, while a different color dot denotes each partnership. Dots with black outlines are outliers.

Torque oscillations over the course of a trial were linearly related to kinematic fluctuations in sway angle based on a mass-spring-damper model (Figures 7A,B). Coefficients from the mass-spring-damper model were all different from zero (*p* < 0.05). Coefficients with positive values correspond to passive mechanical elements that resist motion of the beam-walker’s body while negative values correspond to active elements that amplify motion. Coefficients for Ty_Fx (inertia = 1.7 ± 1.7 kg* m^2^, damping = −21 ± 13 N*m/(rad/s), and stiffness = −64 ± 57 N*m/rad) and Ty_Fz (inertia = −1.4 ± 0.92 kg*m^2^, damping = 13 ± 5.7 N*m/(rad/ s), and stiffness = 76 ± 31 N*m/rad) had opposite signs, reflecting opposite types of mechanical elements (Figure 7C).

## Discussion

This first study quantifying human-human physical interactions in a balance-challenging walking task relevant to physical assistance and rehabilitation suggests that hand interactions aid balance primarily through haptic communication. With a partner, participants were able to walk further on the beam without falling, reduce lateral sway, and decrease angular momentum in the frontal plane. Consistent with haptic communication, hand forces used to aid balance were small and created interaction torques on the beam-walker’s body about the beam axis. Balance improvements with a partner were correlated with the amount of interaction torque, which does not contribute to movement and likely provides haptic communication. Finally, the relationship between torques and motion of the beam-walker’s body can be represented by opposite types of mechanical elements (inertia, damping, and stiffness) that can be implemented in robot controls to aid walking balance.

Our approach examines partner interactions in a paradigm relevant to physical assistance and rehabilitation. The beam-walking task challenges balance in unimpaired young adults while persons with balance impairments may be challenged by overground walking. Notably, there was no correlation between improvement in balance performance and the beam-walker’s solo balance ability, demonstrating that physical interaction benefits participants of varying ability levels. Our results may be relevant to individuals with balance impairments, but the specific disorders may also impair sensorimotor function in different ways and thus the relationships between hand interactions and balance performance must be explicitly tested in impaired populations. For example, proprioceptive and cutaneous sensory acuity and muscular strength may affect the degree to which hand interactions are used for communication or mechanical support.

The small hand force magnitudes (mean of 2.2 N laterally and 3.4 N vertically) we observed during partnered beam-walking may reflect a combination of passive dynamics of moving limbs during walking, light touch, and haptic communication. Although previously a 1 N threshold was established for light touch during standing (Holden et al., 1994; Jeka and Lackner, 1994), there may be additional interaction forces during walking due to the passive dynamics of arm and hand motion without intentional communication or cooperation; hand forces in handholding during treadmill walking (i.e. no balance aid) were previously measured to be within a range of 2–4 N (Sylos-Labini et al., 2018). Therefore, balance improvement during partnered beam-walking as well as during interpersonal light touch in standing balance may arise from similar mechanisms, e.g., increased sensory feedback on the body’s spatial localization (Holden et al., 1994; Jeka and Lackner, 1994; Johannsen et al., 2016). Moreover, the hand forces we observed are slightly lower than during a partnered stepping task that required haptic communication; they found peak interaction forces of ∼7 N between novice partners during an unpredictable forward-backward stepping sequence (Sawers et al., 2017). Although it is arguable what exact amount of force is meaningful for mechanical support in our balance task, the fact that we measured hand forces during balance aid similar to that of handholding during walking without balance aid suggests that the forces primarily served sensory feedback rather than direct mechanical support, and thus can be considered both “light touch” and “haptic communication.”

The opposing torques created by hand forces further support that balance during partnered beam-walking is aided through haptic communication. Prior studies in dyadic object manipulation identified opposing forces that facilitated haptic communication (Reed et al., 2006; Reed and Peshkin, 2008; van der Wel et al., 2011; Sawers et al., 2017; Jensen et al., 2021). Similarly, during partnered beam-walking, we show that orthogonal components of the hand interaction forces cause opposing torques in the plane of body sway during beam-walking, consistent with the rotational dynamics of the body about the beam (Chiovetto et al., 2018). Although each of the torque components (Ty_Fx and Ty_Fz) was higher during partnered beam-walking than partnered overground walking, neither torque magnitude was correlated with balance improvement. There were also non-zero mean net torques that may reflect normal force supporting the weight of the beam-walker’s arm, light touch, and/or haptic communication, but these net torques were not correlated with balance improvement. In contrast, we found that greater interaction torque was correlated with greater reduction in body sway during partnered beam-walking. Our results are consistent with the finding that larger interaction forces result in better performance during a partnered walking task (Sawers et al., 2017). Because interaction torque creates no net torques on the body, it does not contribute directly to body sway, and likely improves balance through haptic communication. The bias we used to account for sensor drift did not qualitatively alter our results. We tested high and low extreme values of sensor drift, for which the difference in opposing torque magnitude between partnered overground *vs.* beam walking (Supplementary Figure S1A) and the correlation between balance improvement and interaction torque magnitude remain significant (Supplementary Figure S1B). Mass-spring-damper model estimates rely on the variance in the signal and would not be affected by the bias.

However, interaction torque could contribute to increased mechanical stability of the beam-walker’s body by increasing the net impedance in a manner similar to co-contraction (Reed and Peshkin, 2008; Melendez-Calderon et al., 2015). In our setup we were not able to explicitly decouple forces and torques used for communication vs task dynamics (impedance). This decoupling is very difficult to achieve in a walking task, but has been done in upper-limb studies with virtual objects that either isolated force feedback from the object *vs*. the partner (Groten et al., 2013; Roche and Saint-Bauzel, 2016) or used physical interaction to negotiate a joint non-motor decision (Pezzulo et al., 2019).

The human-human balance-aiding principle of opposing dynamics for lateral and vertical directions is applicable to many robotic walking aids and rehabilitation devices regardless of exact hardware design. Our modeling results suggest that balance aid from a human partner during walking can be represented by two mass-spring-damper systems with opposite types of elements; torques from lateral forces are consistent with active (i.e., energy-injecting) impedance while torques from vertical forces are consistent with passive (i.e., energy-dissipating) impedance. Lateral forces generally create torques acting in the same direction as the beam-walker’s body motion and may function both to communicate information haptically and mechanically alter balance. Vertical forces may reflect normal forces to counter the mass of the beam-walker’s arm, which is consistent with passive impedance. While passive impedance can be implemented with conventional springs, dampers, and masses, “active impedance” (Aguirre-Ollinger et al., 2007) can only be physically realized with an actuated robotic system. Active impedance devices have been shown to improve performance in lower-limb target-acquisition (Aguirre-Ollinger et al., 2007) and upper-limb exploration (Huang et al., 2010) tasks. Several robotic walking aids use an admittance controller to determine the device’s output velocity based on hand forces through the equation: $F=M\ddot{x}+B\dot{x}$ , where F are forces exerted by the user at the handles, $\ddot{x}$ is acceleration, $\dot{x}$ is velocity, and M and B are inertia and damping matrices (Chuy et al., 2005; Spenko et al., 2006; Nakagawa et al., 2016; Jiang et al., 2017). Notably, our model demonstrates the importance of a stiffness coefficient relative to the balance equilibrium point (vertical torso position) that we have not found implemented in any existing robotic walking aids and warrants further exploration. The coefficient values from our modelling results may be implemented directly in admittance controllers of existing robotic devices to emulate the intuitive balance-aiding strategies used in human-human interaction but are likely more appropriate for certain physical setups. For example, our experimental paradigm is more similar to a robotic cane moving beside the person and operated by one hand than a robotic walker/rollator moving in front of the person and operated by two hands. Finally, the fact that haptic balance aid can be modeled by a mass-spring-damper (and thus implemented in robotic systems) does not imply that the human-human interaction is created by mechanical elements; in fact, the haptic signal may communicate information related to acceleration, velocity, and position.

Our model also has limitations in its ability to fully describe human-human haptic interaction to aid balance. The interactions between humans were quite complex, as participants were free to choose how to interact and could vary force/torque magnitude, direction, and timing. The model has several underlying assumptions - such as linearity and time-invariance–that are not true of biological systems, and more complex nonlinear and transient processes may need to be added in the future to create a more comprehensive model. The inertia coefficient (I) of the model may not be reliable as acceleration was not measured directly, and it may represent a combination of the mass of the force handle, force sensor, and the beam-walker’s arm or body. Considerable variability in model coefficients and goodness of fit likely reflect the complexity of the balance task and variations in strategies between partnerships; e.g., some partnerships may communicate more information related to displacement while others may focus more on velocity. Relevant to design of robotic devices to aid human balance, the amount and type of physical interaction should vary according to the ability and needs of the user. Overall, the model provides a useful conceptual framework that can be tested in robotic systems in the future.

## Data Availability

The datasets presented in this study can be found in online repositories. The names of the repository/repositories and accession number(s) can be found below: https://osf.io/s8gr9/.
