# Supplementary figures and images for: Human-Human Hand Interactions Aid Balance During Walking by Haptic Communication
Source: Front Robot AI. 2021 Nov 4;8:735575. doi: 10.3389/frobt.2021.735575 (PMC8599825; doi:10.3389/frobt.2021.735575)

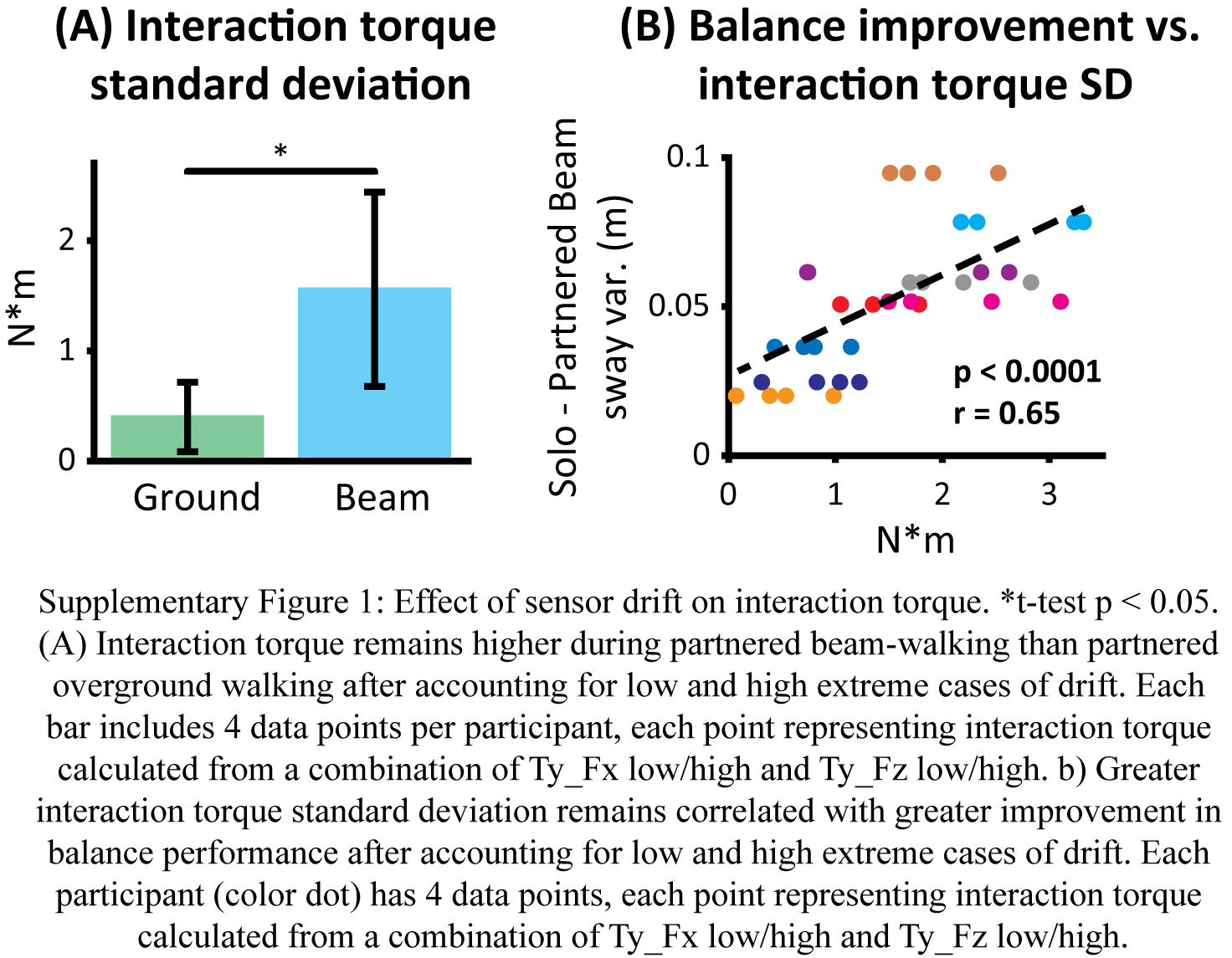

Supplement: Supplementary file 1 [file Image1.TIF]
